# The Prognosis of Urothelial Carcinoma Between Kidney Transplantation Recipients and Normal Patients: A Propensity Score‐Matched Study

**DOI:** 10.1002/cam4.71414

**Published:** 2025-12-09

**Authors:** Wu Taihao, Du Chunkai, Lyu Jingcheng, Zhu Yichen

**Affiliations:** ^1^ Department of Urology Beijing Friendship Hospital, Capital Medical University Beijing China; ^2^ Institution of Urology Beijing Municipal Health Commission Beijing China; ^3^ Department of Urology Peking University International Hospital Beijing China

**Keywords:** kidney transplantation, prognosis, urothelial carcinoma

## Abstract

**Objective:**

To evaluate the difference in the characteristics and prognosis between kidney transplantation (KT) recipients and normal patients with urothelial carcinoma.

**Method:**

We retrospectively reviewed the characteristics of the kidney‐transplanted patients and the general patients who were diagnosed with urothelial carcinoma between 2013 and 2021. The non‐kidney transplantation (NKT) group is matched with the KT group through propensity score matching (PSM) to adjust for confounders. We evaluated the prognosis including overall survival (OS), relapse‐free survival (RFS), and progression‐free survival (PFS). We compared the difference in the prognosis between the two groups using Kaplan–Meier analysis and the log‐rank test.

**Result:**

The characteristics of 344 NKT patients and 42 KT recipients with urothelial carcinoma were included in this research. The percentage of females was significantly higher in the KT group than in the NKT group (*p* < 0.001). Tumors mostly occurred in the upper tract in the KT group and in the bladder cancer (BC) in the NKT group. After propensity score matching (PSM), overall survival (OS) was significantly longer in the KT group with upper tract urothelial carcinoma (UTUC) than in the NKT group (*p* = 0.001), whereas recurrence‐free survival and progression‐free survival (PFS) were significantly lower in the KT group with UTUC+BC than in the NKT group (*p* = 0.028, 0.044).

**Conclusion:**

Patients with tumors that occur in both the bladder and upper urinary tract have a worse prognosis. Kidney transplantation recipients with only bladder or upper urinary tract UC have a prognosis that is no worse or even better than that of NKT patients.

Malignant tumor is a frequent and significant health concern following kidney transplantation (KT). Urothelial carcinoma (UC) is the most common urological malignant tumor in kidney transplant (KT) recipients. The incidence of UC in kidney transplant (KT) recipients is approximately three times higher than that in the general population [1, 2]. The incidence of UC is particularly high in East Asia. This is mainly attributed to aristolochic acid. Aristolochic acid causes permanent mutations in p53, leading to the development of tumors and renal failure [3, 4, 5]. Although it is now generally recognized that the prognosis of this group is worse than that of the general population, there are few articles reporting the tumor recurrence and long‐term survival of patients in this group. Therefore, we retrospectively analyzed the incidence of UC in the KT population and non‐KT population diagnosed in our center from January 2013 to December 2021 and compared their prognosis between the two groups.

## 1 Method

### 1.1 Study Approval and Patient Consent

The protocol for this research project has been approved by a suitably constituted Ethics Committee of the institution, and it conforms to the provisions of the Declaration of Helsinki, Committee of Beijing Friendship Hospital, Approval No. BFH20240116001. The requirement for informed patient consent was waived due to the retrospective nature of this study.

### 1.2 Study Population

This retrospective study included all patients who were diagnosed with UC in our center between January 2013 and December 2021. These patients were divided into the Kidney Transplant (KT) group and Non‐Kidney Transplantation (NKT) group. The KT group included only recipients with stable kidney function, meaning they had normal serum creatinine levels (Scr) during long‐term follow‐up and showed no signs of issues like abnormal urine output, edema, or nausea. Patients with graft dysfunction or impaired transplanted kidney function were excluded. Patients only with Scr in the normal range were included in the NKT group.

All patients in the KT group had tumors in their native urinary system, not the transplanted ones. We also excluded patients with other malignancies or incomplete medical records. Only those who chose surgical treatment were included, while those who opted for other treatments were excluded. Also, we excluded patients with lymphatic metastases and distant metastases. Finally, patients with serious comorbidities such as cardiac valve replacement were excluded.

### 1.3 Definitions

Overall survival (OS) was defined as the time to all‐cause death. Relapse‐free survival (RFS) was defined as the time to recurrence. Progression‐free survival (PFS) was defined as the time to metastasis to any other site.

### 1.4 Statistical Analysis

We analyzed the differences in oncological characteristics between the KT and NKT groups according to the location of tumor occurrence respectively. Then we matched the two groups of patients who developed in the bladder and upper urinary tract separately by Propensity Score Matching (PSM) and analyzed their characteristics again. After that, we analyzed the OS, RFS, and PFS of the two groups of patients whose two sites had been paired and all the patients who developed in both the bladder and the upper urinary tract, respectively.

Categorical variables were compared using Fisher's exact test or the chi‐square test and are presented as numbers and percentages. Continuous variables with normal distributions were compared with the t‐test and are presented as the mean ± standard deviation, while continuous variables with non‐normal distributions are presented as the median and interquartile range. The Kolmogorov–Smirnov test was used to assess the normality of the distribution of continuous variables. Kaplan–Meier analysis and the log‐rank test were used to evaluate OS, RFS, and PFS.

We also performed a post hoc power analysis using G Power software to evaluate the statistical power of our study on the UTUC group. We input the hazard ratio (HR) of 0.379, a significance level (α) of 0.05, and the sample size of 48. The analysis showed a statistical power of 0.73.

## 2 Result

### 2.1 Baseline

We compared the characteristics between the KT group and the NKT group as shown in Table 1. The KT group has significantly more women (*p* < 0.001) and more smoking people (*p* = 0.001). The KT group is more likely to occur in the upper urinary tract, while the NKT group is more likely to occur in the bladder, which two groups have significant differences (*p* < 0.001). The tumors in the KT group are more multifocal (*p* = 0.005). There is no significant difference between the two groups in the T stage (*p* = 0.391) and tumor grade (*p* = 0.589).

**TABLE 1:** Characteristics of KT and NKT groups.

	NKT	KT	*p*
N	344	42	
Gender			< 0.001
Male	233	12	
Female	111	30	
Smoking			0.001
Yes	96	2	
No	248	40	
Site of tumor			< 0.001
BC	214	5	
UTUC	118	29	
BC + UTUC	12	8	
T Stage			0.391
T1	228	23	
T2	59	9	
T3	54	10	
T4	3	0	
High grade			0.589
Yes	104	11	
No	240	31	
Multifocal			0.005
Yes	113	23	
No	231	19	

We also analyzed the characteristics between the two groups in different sites respectively as shown in Table 2. In patients with BC and BC + UTUC, all characteristics between the two groups have no significant difference (*p* > 0.05). Among the patients with UTUC, the KT group has a higher percentage of females than the NKT group (*p* = 0.012). KT group tumors are more multifocal (*p* < 0.001).

**TABLE 2:** Characteristics of KT and NKT groups divided by different sites.

Characteristics	NKT	KT	*p*
*BC* *			
N	214	5	
Gender: Male	170	4	
T stage			
T1	177	4	
T2	25	1	
T3	10	0	
T4	2	0	
High grade	144	4	
Multifocal	89	2	
Bladder infusion	150	2	
GC/GCa chemotherapy	21	1	
*UTUC*			
N	118	29	
Gender: Male	59	7	0.012
T Stage			0.743
T1	45	14	
T2	31	7	
T3	41	8	
T4	1	0	
High grade	87	19	0.377
Multifocal	12	15	< 0.001
Bladder infusion	48	9	0.340
GC/GCa chemotherapy	29	7	0.961
*BC + UTUC*			
N	12	8	
Gender: Male	4	1	0.603
T Stage			0.774
T1	6	5	
T2	3	1	
T3	3	2	
T4	0	0	
High grade	9	8	0.065
Multifocal	12	6	0.147
Bladder infusion	5	7	0.070
GC/GCa chemotherapy	4	3	> 0.999

* The *p*‐value lacks significance due to the insufficient sample size.

KT group and NKT group with BC or UTUC tumors are matched through the PSM, and each group has 5 and 24 patients for BC and UTUC. The patients with BC + UTUC are not matched due to the similar count and characteristics. The characteristics between the two groups that have been matched are shown in Table 3. No significant differences in all characteristics of the matched patients except age at UTUC (*p* > 0.001). Because of the large age difference between the two groups, there was still a difference between the two groups in terms of age after matching. We also compared the characteristics of kidney‐transplanted patients and found no significant difference (Table 4).

**TABLE 3:** The characteristics after PSM matched.

Characteristics	KT	NKT	*p*
*BC*			
N	5	5	
Age	60.6 ± 16.59 (40.00–81.20)	76.2 ± 4.55 (70.55–81.85)	0.077
Male	4 (80%)	5 (100%)	0.292
Smoke	4 (80%)	4 (80%)	> 0.999
T Stage			> 0.999
T1	4 (80%)	4 (80%)	
T2	1 (20%)	1 (20%)	
High grade	4 (80%)	3 (60%)	0.49
Multifocal	2 (40%)	3 (60%)	0.527
Bladder infusion chemotherapy	2 (40%)	4 (80%)	0.197
GC/GCa chemotherapy	1 (20%)	0 (0%)	0.292
*UTUC*			
	24	24	
Male	7 (29.2%)	8 (33.3%)	0.755
Smoke	1 (4.2%)	4 (16.7%)	0.156
Side			0.463
Left	12 (50.0%)	10 (41.7%)	
Right	11 (45.8%)	14 (58.3%)	
Both	1 (4.2%)	0 (0%)	
T Stage			0.747
T1	10 (41.7%)	10 (41.7%)	
T2	7 (29.2%)	5 (20.8%)	
T3	7 (29.2%)	9 (37.5%)	
High grade	16 (66.7%)	15 (62.5%)	0.763
Multifocal	10 (41.7%)	10 (41.7%)	> 0.999
Bladder infusion chemotherapy	7 (29.2%)	8 (33.3%)	0.755
GC/Gca chemotherapy	7 (29.2%)	5 (20.8%)	0.505
Age	56.63 ± 9.53 (52.60–60.65)	70.71 ± 8.94 (66.93–74.48)	< 0.001

**TABLE 4:** The characteristics of KT group patients in different sites.

Characteristics	BC (*N* = 5)	UTUC (*N* = 24)	UTUC+BC (*N* = 8)	*p*
Sirolimus	0 (0%)	4 (16.7%)	1 (12.5%)	0.609
Tacrolimus	2 (40%)	9 (37.5%)	3 (37.5%)	0.994
CsA	3 (60%)	10 (41.7%)	5 (62.5%)	0.512
The time between trans and tumor (month)	135.6 ± 120.6 (−14.1–285.3)	144.5 ± 42.5 (126.6–162.4)	128.1 ± 53.2 (83.67–172.6)	> 0.05*

* *p* = 0.878 (BC vs. UTUC); 0.382 (UTUC vs. UTUC+BC); 0.901 (BC vs. UTUC+BC).

### 2.2 Survival

**FIGURE 1:**
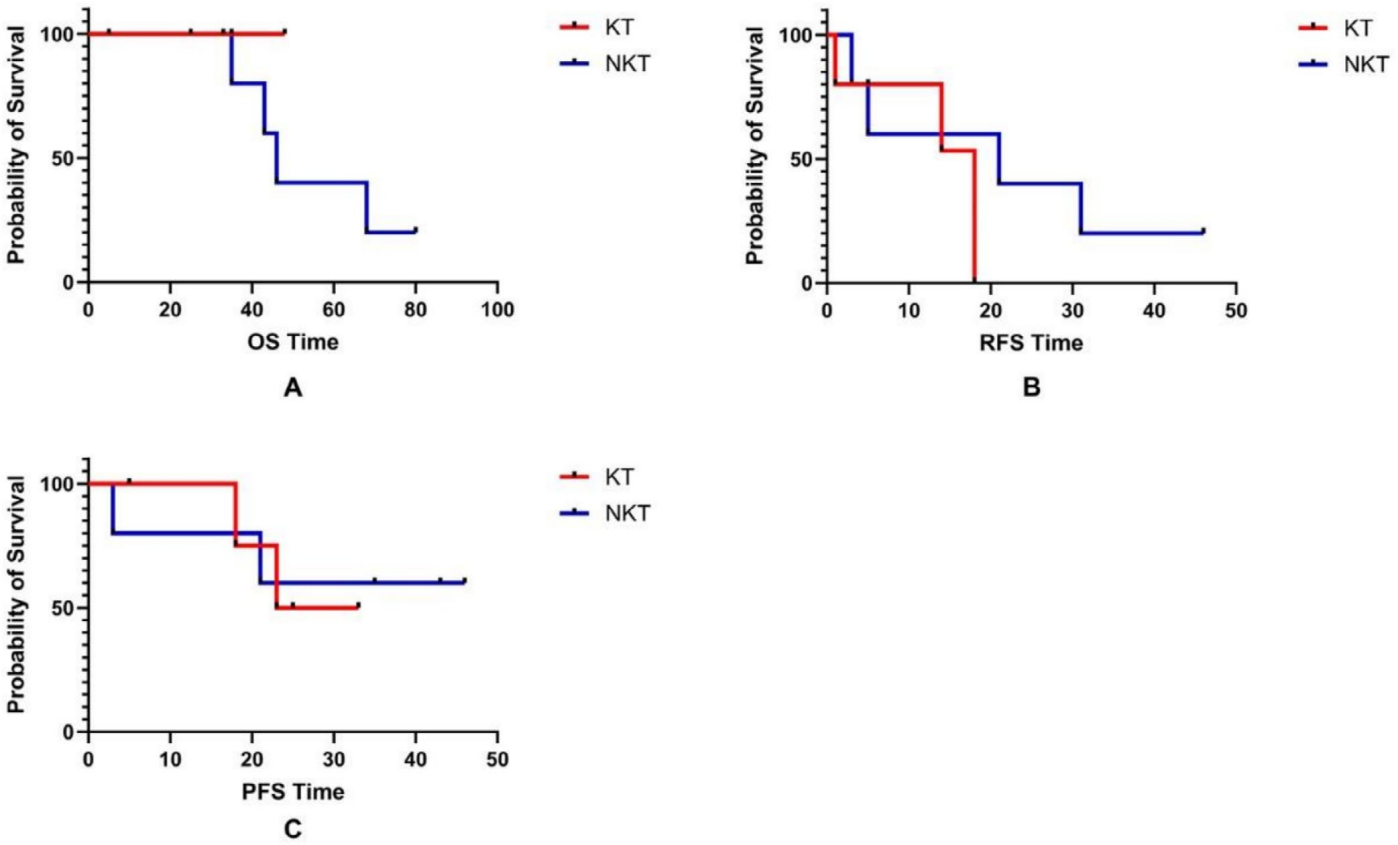
The prognosis of the patients in different groups with BC. A: The difference in OS between the KT recipients and NKT patients with BC; B: The difference in RFS between the KT recipients and NKT patients with BC; C: The difference in PFS between the KT recipients and NKT patients with BC.

We compared the prognosis of patients who developed BC in both groups, as shown in Figure 1. Due to the few patients in the two groups, the comparison between the KT and NKT groups will not have a statistically significant difference, so we did not specifically analyze the OS, RFS, and PFS. The average PFS time in the KT group and NKT group is 26.75 ± 3.25 (20.39–33.12) months and 32.40 ± 7.87 (16.97–47.83) months, respectively.

**FIGURE 2:**
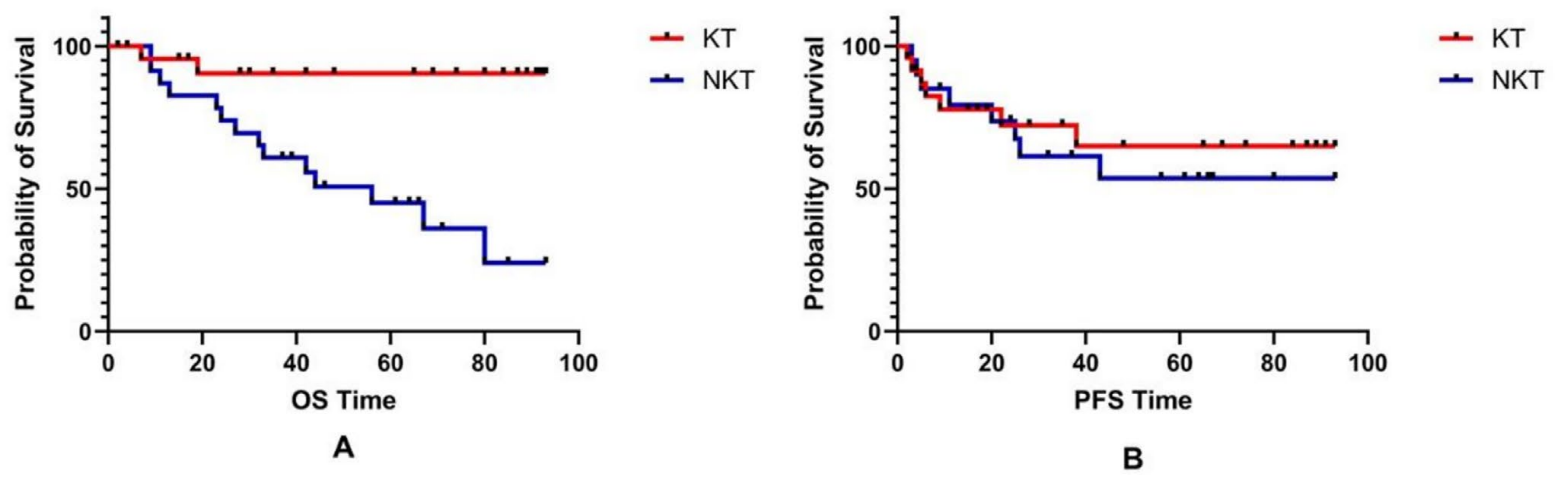
The prognosis of the patients in different groups with UTUC. A: The difference in OS between the KT recipients and NKT patients with UTUC; B: The difference in PFS between the KT recipients and NKT patients with UTUC.

We compared the prognosis of patients who developed UTUC in both groups, as shown in Figure 2. The OS of the KT group is significantly higher than that of the NKT group (*p* = 0.001). The five‐year survival rates of the KT and NKT groups are 90.4% and 45.1%, respectively. The mean OS time of the KT group is 85.37 ± 5.14 (75.31–95.44) months, and the NKT group is 53.41 ± 6.76 (40.17–66.65) months. The PFS between the two groups has no significant difference (*p* = 0.467). The mean PFS time of the KT group is 69.50 ± 8.22 (53.39–85.60) months, and the NKT group is 58.74 ± 9.15 (40.80–76.68) months.

**FIGURE 3:**
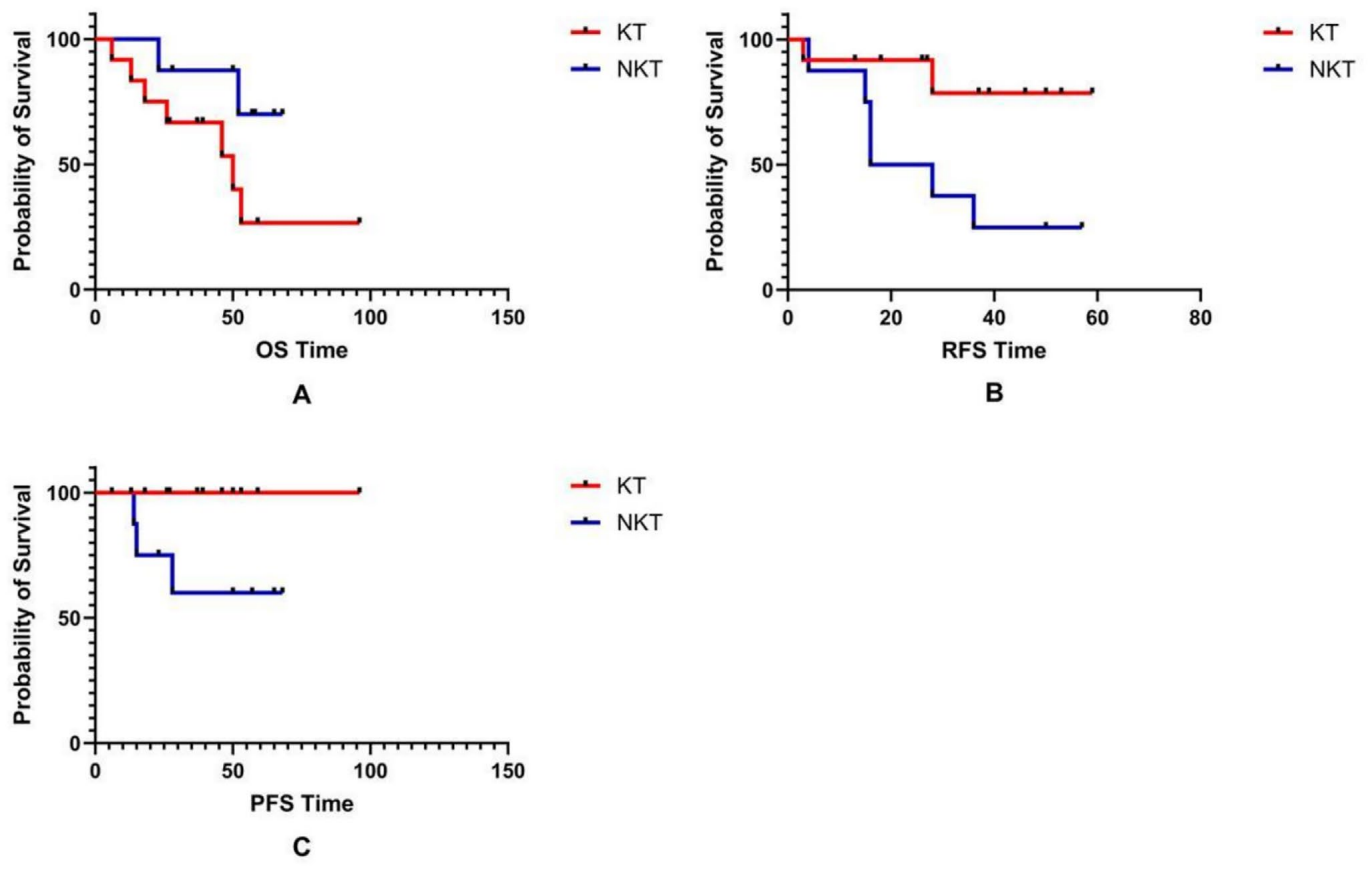
The prognosis of the patients in different groups with BC + UTUC. A: The difference in OS between the KT recipients and NKT patients with BC + UTUC; B: The difference in RFS between the KT recipients and NKT patients with BC + UTUC; C: The difference in PFS between the KT recipients and NKT patients with BC + UTUC.

We compared the prognosis of patients who developed BC + UTUC in both groups, as shown in Figure 3. The OS between the two groups has no significant difference (*p* = 0.089). The five‐year survival rates of the KT group and NKT group are 70.0% and 26.7%, respectively. The mean OS time of the KT group is 59.58 ± 5.49 (48.81–70.34) per month, and the NKT group is 50.72 ± 9.93 (31.25–70.19) per month. The RFS and PFS of the KT group are significantly lower than those of the NKT group (*p* = 0.028, 0.044). The mean RFS time of the KT group is 28.63 ± 6.59 (15.72–41.53) months, and the NKT group is 50.27 ± 5.57 (39.35–61.20) months.

## 3 Discussion

Malignant tumor is a common long‐term health issue after KT, occurring at a significantly higher rate than in the general population. Among these tumors, UC stands out as the most frequent urological malignancy in KT recipients, with an incidence rate approximately three times greater than that in the general populace. This increased prevalence is largely attributed to prolonged immunosuppression post‐transplantation and polyomavirus infection. In East Asia, KT recipients face a significantly heightened risk of developing UC, primarily due to exposure to aristolochic acid, a known carcinogen in this population [1]. Aristolochic acid is capable of inducing mutations from A: T to T: A, thereby fostering the development of UC, as well as causing renal function impairment and, in severe cases, failure [4, 5].

The primary approach to treating UC following KT is surgical intervention. This is because systemic chemotherapy or immunotherapy is infrequently employed in the KT population due to concerns regarding nephrotoxicity and graft rejection. In our previous study [6], we determined that while chemotherapy using the GC/GCa regimen is deemed safe for KT recipients, it does not significantly influence patient prognosis. Other studies have shown that for UTUC patients without metastases, the impact of adjuvant and neoadjuvant chemotherapy on survival still requires further clinical evidence [7, 8]. This is why we did not conduct an in‐depth analysis of chemotherapy use. Furthermore, our research revealed that recipients with UC, regardless of whether it affected the upper urinary tract or bladder, exhibited a more favorable prognosis. Therefore, in the present study, we conducted an analysis to compare the prognosis between the KT group and the NKT group.

Kidney transplant (KT) recipients with urothelial carcinoma (UC) exhibited a higher proportion of females and a greater incidence of upper tract urothelial carcinoma (UTUC). Upon further comparison with the non‐kidney transplant (NKT) population, we noted that gender differences were predominantly observed in patients who developed UTUC. Furthermore, KT recipients with UTUC were more prone to having multiple tumors, a finding consistent with the majority of other studies [9]. This characteristic is primarily linked to a history of exposure to aristolochic acid [10, 11]. Additionally, we posit that it may also be associated with long‐term immune system suppression.

Our analysis revealed no significant difference in tumor T‐stage and grade between KT recipients and NKT patients, regardless of the tumor's site. This suggests that KT does not influence the timing of tumor‐related symptom detection in patients.

Furthermore, we observed a significant age difference between KT recipients with UTUC and NKT patients, a difference that persisted even after PSM. This discrepancy is primarily attributed to the younger age of KT recipients at the time of transplantation and the limited survival time of most transplanted kidneys. Consequently, the age of patients in the KT group at the onset of tumor development was more constrained due to the inclusion criteria of this study. In contrast, no such constraints existed for patients in the NKT group, resulting in a substantial age gap between the two cohorts. This age difference may influence patient prognosis, potentially leading to a more favorable prognosis in the younger KT group [12, 13].

For patients with BC, no differences were observed between the KT and NKT groups in terms of oncological characteristics. Due to the small sample size, no significant differences were observed between the groups in this part of the analysis, and thus additional comparisons were not pursued.

In patients with UTUC, the OS of recipients in the KT group was higher than that in the NKT group, which is challenging to explain. Upon analyzing the use of immunosuppressive drugs in the KT group, we found that rapamycin was utilized in less than 20% of patients, alongside antiproliferative drugs and glucocorticosteroids. Therefore, we believe that the superior survival rate in the KT group is not attributable to immunosuppressive drugs, particularly rapamycin. Instead, we speculate that this higher survival rate may be linked to the performance of contralateral prophylactic nephrectomy and ureteral resection in some of our patients. Additionally, regular monitoring of urinary routine in KT patients may facilitate the prompt detection of early microscopic hematuria, leading to earlier treatment compared to the NKT group.

In patients with BC + UTUC, no disparity in survival was observed between the KT and NKT groups. However, KT recipients were more prone to recurrence and metastasis. We attribute this tendency to the heightened malignancy of this tumor subgroup, leading to more pronounced differences between the two groups. Furthermore, this disparity may also be linked to the immunosuppression maintained in the KT group.

In this study, we observed no significant difference in prognosis, particularly in terms of patient survival, between the two groups. Both the KT and NKT groups exhibited a higher survival rate, a finding consistent with the results of most other studies [9, 14]. One study indicated that patients with UC caused by aristolochic acid generally had a better prognosis [15]. Additionally, the degree of immunosuppression gradually decreases after KT due to the development of immune tolerance to the transplanted kidney by the immune system. This reduction in immunosuppression is maintained, on average, for about ten years from transplantation to the development of tumors. Therefore, we believe that the lower level of immunosuppression is also a contributing factor to the better prognosis.

There are several limitations in this article. Firstly, the sample size of kidney transplant (KT) recipients is insufficient. The small sample size of some KT group recipients after grouping could reduce the credibility of the findings. However, due to the specificity of KT recipients, we included all eligible patients during this time period. Secondly, this paper only compares the prognosis of the KT group with that of the general population with normal renal function, lacking a comparison with other groups of patients who received renal replacement therapy or experienced graft loss. This limitation hinders our ability to accurately understand the prognosis and differences among end‐stage renal disease groups with urothelial carcinoma receiving different renal replacement therapies. Finally, while propensity score matching (PSM) was used to adjust for confounders between kidney transplant (KT) recipients and non‐transplant (NKT) patients, it's important to note that KT is a clinical decision, not a patient choice. This decision is influenced by various individual factors that may also affect the prognosis of urothelial carcinoma (UC). As a result, while PSM enables statistical comparisons, the clinical relevance of these findings may be limited by the factors guiding KT selection.

In conclusion, the prognosis was similar between the KT group and the NKT group with BC only or UTUC. However, in patients with tumors occurring in both the bladder and upper urinary tract, the prognosis was worse in the KT group.

## Author Contributions

**Wu Taihao:** data curation (lead), formal analysis (equal), investigation (lead), writing – original draft (lead). **Du Chunkai:** formal analysis (equal), methodology (lead), visualization (lead). **Lyu Jingcheng:** conceptualization (lead), validation (lead), writing – review and editing (lead). **Zhu Yichen:** funding acquisition (lead), project administration (lead), supervision (lead).

## Funding

This work was supported by the Beijing Key Clinical Specialty Project 20240930 and the National Key Clinical Specialty Project 20250829.

## Ethics Statement

The protocol for this research project has been approved by a suitably constituted Ethics Committee of Beijing Friendship Hospital, Approval No. BFH20240116001, and it conforms to the provisions of the Declaration of Helsinki. The requirement for informed patient consent was waived due to the retrospective nature of this study. The Ethics Committee of Beijing Friendship Hospital waived the requirement for informed patient consent.

## Conflicts of Interest

The authors declare no conflicts of interest.

## Data Availability

I confirm that my article contains a Data Availability Statement even if no data is available (list of sample statements) unless my article type does not require one. I confirm that I have included a citation for available data in my references section, unless my article type is exempt.
